# Impact of near-infrared fluorescence imaging with indocyanine green on the surgical treatment of pulmonary masses in dogs

**DOI:** 10.3389/fvets.2023.1018263

**Published:** 2023-02-07

**Authors:** Naoki Sakurai, Kumiko Ishigaki, Kazuyuki Terai, Tatsuya Heishima, Kazuki Okada, Orie Yoshida, Yumiko Kagawa, Kazushi Asano

**Affiliations:** ^1^Laboratory of Veterinary Surgery, Department of Veterinary Medicine, College of Bioresource Sciences, Nihon University, Fujisawa, Japan; ^2^North Lab, Sapporo, Japan

**Keywords:** dog, fluorescence, indocyanine green, pulmonary masses, lymph node

## Abstract

**Objectives:**

To investigate the intraoperative identification and complete resection of pulmonary masses, and to evaluate lymph node metastasis of pulmonary malignant tumors in dogs using indocyanine green (ICG) fluorescence imaging.

**Methods:**

Forty dogs with pulmonary masses were included, all of which underwent surgical treatment. ICG fluorescence imaging was performed on pulmonary masses before lobectomy and the resection margins after lobectomy. In addition, ICG fluorescence of the excised masses and lymph nodes was evaluated in the shaded box. The fluorescence findings were compared with the histopathological diagnosis.

**Results:**

Of 44 nodules resected from 40 dogs, 32 nodules were histopathologically diagnosed as lung adenocarcinoma, five were histiocytic sarcoma, three were undifferentiated sarcoma, two were malignant epithelial tumor metastases, one was carcinosarcoma, and one was a non-neoplastic lesion. Fluorescence was observed in all nodules. In addition to the main lesion, other fluorescent nodules were found in four dogs. Regarding the diagnostic accuracy of complete resection based on ICG fluorescence, the sensitivity was 67.7% and the specificity was 60.0%. The sensitivity and specificity of ICG fluorescence for the diagnosis of lymph node metastasis were 100 and 75.0%, respectively.

**Conclusions:**

ICG fluorescence imaging might be a useful intraoperative diagnostic method to identify the location of tumors and lymph node metastasis, but not to evaluate complete tumor resection, in dogs with pulmonary malignant tumors.

## 1. Introduction

Indocyanine green (ICG) is a cyanine fluorescent dye that emits light that peaks at approximately 835 nm when illuminated with near-infrared light (750–810 nm) (1). Intravenously administered ICG is selectively taken up by hepatocytes and excreted in bile without being subjected to metabolism, enterohepatic circulation, or renal excretion. In human medicine, it has been recently shown that near-infrared fluorescence imaging using ICG has various applications, such as intraoperative identification and complete resection evaluation of hepatocellular carcinoma (2–4); identification of sentinel lymph nodes in various cancers such as breast, gastric, lung, and esophageal cancers (5–8); and patency of the graft for coronary artery disease (9). In veterinary medicine, the ICG fluorescence method has been used for identification of 12 nodules of hepatocellular carcinoma in six dogs (10), identification of the thoracic duct in 15 dogs with chylothorax (11), examination of the intraoral sentinel lymph node in six hound dogs (12), intraoperative identification of epithelial bodies in three mongrel dogs (13), vascular visualization in caudal auricular flaps in two cats (14), and angiography for examining the normal ocular fundus in eight dogs (15). Nevertheless, several aspects of ICG imaging remain unclear owing to the relatively small number of reported studies and their small sample sizes.

Primary pulmonary tumors account for 1% of all tumors in dogs (16). Most primary pulmonary tumors are malignant and are most often bronchial or alveolar tumors; others include squamous cell carcinoma, histiocytic sarcoma, and anaplastic tumors (16–18). Surgical treatment is generally preferred for primary or metastatic tumors that are isolated (19). The median survival time (MST) for primary pulmonary tumors in dogs after surgical treatment is reported to be 361 days (18). It has also been reported that the MST of dogs with metastasis to the tracheobronchial lymph nodes is 26 days, while the MST of dogs without lymph node metastasis is significantly longer at 452 days (18). In another study, lymph node metastasis was identified as a prognostic factor (20). These data demonstrate the importance of determining the degree of infiltration and diagnosing lymph node metastasis when considering the prognosis of pulmonary tumors. The purposes of this study were to investigate the intraoperative identification and complete resection of pulmonary masses, and to evaluate lymph node metastasis of pulmonary malignant tumors in dogs using ICG fluorescence imaging.

## 2. Materials and methods

### 2.1. Animals and preoperative examination

This clinical case series study included 40 privately-owned dogs with pulmonary masses at the Animal Medical Center of Nihon University from November 2014 to January 2022. Informed consent was obtained from all owners, and information about all procedures was provided to the owners. All dogs underwent physical examination, blood count, serum chemistry tests, chest radiography, computed tomography (CT), and ICG fluorescence imaging during and after surgery. Furthermore, in dogs where ICG fluorescence imaging was performed on the resected tracheobronchial lymph node, the size of the same node was evaluated from the preoperative CT images. Resected tissues obtained from all cases were used for the histopathological diagnosis. The occurrence of adverse events related to ICG administration during the intra- and postoperative periods was evaluated. No cases were excluded due to missing data in this study.

Informed consent was obtained from all the owners and information about all the procedures were provided to the owners. All the procedures were approved by the Ethical Committee of Nihon University Animal Medical Center.

### 2.2. ICG fluorescence imaging

**Figure 1:**
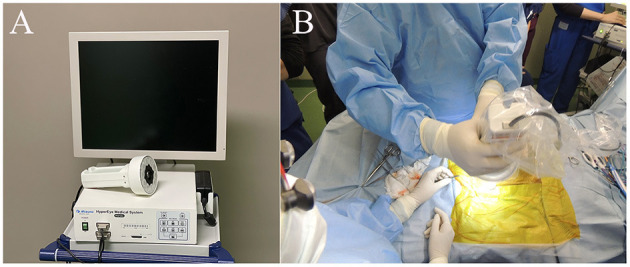
Near-infrared (NIR) fluorescence imaging using the HyperEye Medical System (HEMS). **(A)** Real-time NIR fluorescent images of the pulmonary surface observed on the HEMS monitor. **(B)** Intraoperative observation of a pulmonary fluorescent lesion. The camera unit was equipped with a light-emitting diode (LED) and charge-coupled device (CCD).

Approximately 12–24 h before surgery, ICG (Diagnogreen; Daiichi Sankyo Co. Ltd., Tokyo, Japan) was diluted to 5 mg/mL with distilled water for injection, and 2.0 mg/kg was administered intravenously. During surgery, pulmonary masses were observed visually in the lobes of the lungs exposed by thoracotomy. For the confirmed pulmonary masses, the lung surface was observed with an infrared camera system (HyperEye Medical System; Mizuho Medical Co. Ltd, Tokyo, Japan) before surgical resection (Figure 1). When images were taken, the surgical light was turned off and images were taken at a distance of 30–50 cm from the lung surface. Regardless of the fluorescent findings, pulmonary masses and lymph nodes, for which surgical resection was indicated, were removed. The resection margins of the pulmonary masses in the body were also imaged under the same conditions. The excised masses and lymph nodes were imaged under the same conditions in a shaded box, and the fluorescence was compared.

The fluorescence intensity was defined as 1 when it was stronger than the surrounding normal lung tissue, 0 when it was equivalent, and −1 when it lacked fluorescence. A score of 1 was defined as “fluorescent”, while 0 and −1 were defined as “no fluorescence”.

### 2.3. Histopathological diagnosis

Tissue samples were immersed in 10% neutral buffered formalin for 48 h and then embedded in paraffin. After the sections were deparaffinized with xylene, they were immersed in ethanol. The slides were stained with hematoxylin and eosin and subjected to histopathological testing. The histopathological findings were then compared with fluorescence findings.

### 2.4. Statistical analyses

The sensitivity, specificity and accuracy of complete resection using ICG fluorescence imaging were calculated for the cases in which fluorescence of the resected margin was evaluated. In addition, the chi-square test was used to analyze the relationship between ICG fluorescence of the resected margin and complete resection. Similarly, for cases in which lymph node fluorescence was evaluated, the sensitivity, specificity and accuracy of regional lymph node metastasis detection by ICG fluorescence imaging were calculated, and the chi-square test was performed to analyze the relationship between ICG fluorescence and histopathology of metastasis in the regional lymph nodes. The cutoff value of the size of the tracheobronchial lymph node in CT imaging was set to 12 mm based on a previous report (21). Accordingly, the sensitivity, specificity and accuracy of CT imaging for detection of tracheobronchial lymph node metastasis were calculated, and the chi-square test was used to analyze the relationship between CT findings and histopathology of metastasis in the tracheobronchial lymph nodes. SPSS Statistics 27 (IBM, Brussels, Belgium) was used for the analysis. Statistical significance was set at *p* < 0.05.

## 3. Results

The median age of the dogs was 12.5 year-old [range: 6.4–15.7], and their median body weight was 7.8 kg [range: 1.9–36.3]. There were 19 spayed females, one intact female, 17 castrated males, and three intact males. The 40 dog breeds are summarized in Table 1. The median time from preoperative CT scan to surgery was 6 days [range: 0–41]. The median maximum diameter of the pulmonary masses on CT was 43.1 mm [range: 8.8–165.1].

**Table 1:** Breeds of cases.

**Breed**	**Number of cases**
Miniature Dachshunds	9
Pembroke Welsh Corgis	6
Miniature Schnauzers	3
Border Collies	3
Mixed Breeds	3
Toy Poodles	2
French Bulldogs	2
Labrador Retrievers	2
American Cocker Spaniel	1
Golden Retriever	1
Bernese Mountain Dog	1
Pekingese	1
Shih Tzu	1
Flat Coated Retriever	1
Beagle	1
Jack Russell Terrier	1
Pomeranian	1
Chihuahua	1

Surgical procedures performed are summarized in Table 2. In one of the dogs that underwent resection of the right caudal lobe, a tumor was found in the left caudal lobe 1,110 days after surgery; therefore, resection of the left caudal lobe was performed.

**Table 2:** Surgical procedures for lung lobectomy.

**Surgical procedures**	**Number of cases**
Resection of the left cranial lobe	12
Resection of the left caudal lobe	10
Resection of the right caudal lobe	5
Resection of the right cranial lobe	3
En bloc resection of the right cranial and middle lobes	3
Left pneumonectomy	2
Resection of the right accessory lobe	2
Resection of the right middle lobe	1
Resection of the right middle lobe and en bloc resection of right caudal and accessory lobes	1
Resection of the left cranial lobe and right accessory lobe	1

A total of 44 nodules were resected from 40 dogs with pulmonary masses. Histopathologically, 32 nodules were diagnosed as lung adenocarcinoma, five were histiocytic sarcoma, three were undifferentiated sarcoma, two were malignant epithelial tumor metastases, and one was a carcinosarcoma. One nodule was a non-tumoral lesion, and histopathology showed a collection of foamy macrophages.

**Figure 2:**
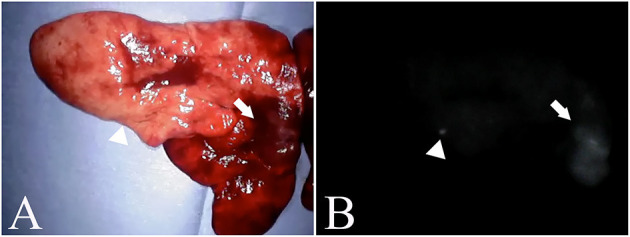
Nodules found outside the main lesion. **(A)** is an excised pulmonary histiocytic sarcoma. **(B)** is a fluorescent image of a similar lesion. The arrow indicates the main lesion, and the arrowhead indicates the nodule. In the nodules, calcification and vasodilation are visible.

The ICG fluorescence imaging was feasible in all dogs. In addition, all dogs showed no complications related with ICG fluorescence imaging. Each main lung mass in all dogs had a score of 1 for fluorescence intensity. Additional fluorescent nodules apart from the main lung mass were observed in four dogs (Figure 2). Of the four dogs, two dogs with lung adenocarcinoma as a main lesion had another fluorescent nodule: the nodule was metastasis of adenocarcinoma in one dog, but a non-tumoral lesion in another dog. One dog with histiocytic sarcoma as a main lesion had another fluorescent nodule which was a non-tumoral lesion, and the other dog had a large metastatic lung mass from mammary tumors as a main lesion and four other metastatic fluorescent nodules. In the four dogs with the fluorescent nodules apart from the main lesion, the contrast-enhanced CT revealed both nodules in two dogs with lung adenocarcinoma and three out of four nodules in the dog with metastases from the mammary tumors. In contrast, the residual one nodule in the dog with metastasis from the mammary tumors and one nodule in the dog with histiocytic sarcoma were not detected by the contrast-enhanced CT. The non-tumoral fluorescent nodules apart from the main lesion included calcification, ossification, and dilation of the blood vessels revealed by the histopathological examination.

**Figure 3:**
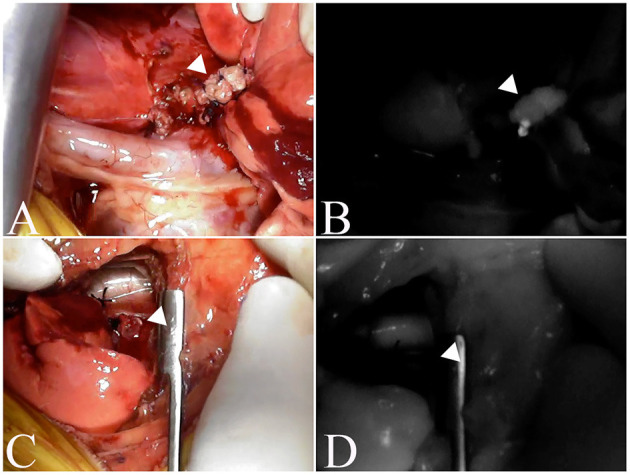
Fluorescence of the excised margin after excision of the tumor. The arrowhead indicates resection margins. **(A, C)** are the resection margins of lung adenocarcinoma, and **(B, D)** are fluorescence images of the resection margins of the same case. **(A, B)** is an incomplete resection where fluorescence is observed, whereas **(C, D)** is a complete resection and no fluorescence is observed.

In 23 nodules, fluorescence of the resection margin was compared with the evaluation of the surgical margin, and two of the three nodules with incomplete resection showed fluorescence, while one nodule did not (Figure 3). Of the 20 nodules with complete resection, eight nodules showed fluorescence, while 12 nodules did not. The sensitivity, specificity and accuracy of ICG fluorescence for complete resection of pulmonary tumors were found to be 66.7, 60.0, and 60.9%, respectively. There was not a significant relationship between ICG fluorescence of the resected margin and complete resection (*p* = 0.3849).

Lymphadenectomy was performed in 11 dogs, and 13 lymph nodes were removed. Of these, 11 were tracheobronchial lymph nodes and two were cranial mediastinal lymph nodes. Histopathologically, metastatic lesions were found in five of the tracheobronchial lymph nodes.

**Figure 4:**
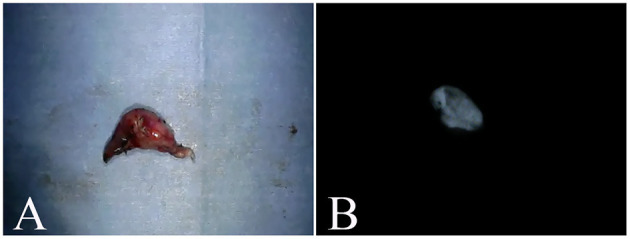
Excised tracheobronchial lymph nodes. **(A)** is a tracheobronchial lymph node from a case of metastasis from a malignant epithelial tumor. **(B)** is the fluorescent image of a similar lymph node, with confirmed fluorescence.

Seven lymph nodes (six tracheobronchial lymph nodes and one cranial mediastinal lymph node) resected from six dogs whose lymph node fluorescence was evaluated were compared in terms of the presence of metastasis. Fluorescence was observed in all three nodes with metastasis, whereas fluorescence was observed in only one of four nodes without metastasis; no fluorescence was observed in the remaining three nodes (Figure 4). The sensitivity, specificity and accuracy of ICG fluorescence for evaluating lymph node metastasis of pulmonary tumors were found to be 100, 75.0, and 85.7%, respectively. There was a significant relationship between ICG fluorescence and histopathological metastasis of the lymph nodes (*p* = 0.0472).

The size on CT images and fluorescence of tracheobronchial lymph nodes are summarized in Table 3. The median size of the six tracheobronchial lymph nodes evaluated for fluorescence on CT images was 8.0 mm [range: 4.6–18.3]. For these cases, the median maximum diameter of the pulmonary masses on CT images was 35.4 mm [range: 30.1–42.0]. When the cutoff value of the tracheobronchial lymph node size was set at 12 mm, the sensitivity, specificity and accuracy of the diameter of the pulmonary mass on CT for detection of lymph node metastasis was 33.3, 100, and 66.7%, respectively. There was not a significant relationship between CT findings and histopathological metastasis of the lymph nodes (*p* = 0.2733).

**Table 3:** Size on CT images and fluorescence of tracheobronchial lymph nodes.

**Lymph node no**.	**Histopathological diagnosis of main lesion**	**Metastasis**	**Size on CT images**	**Fluorescence**
			**(mm)**	
#1	Lung adenocarcinoma	No	6.9	No
#2	Lung adenocarcinoma	No	7.8	No
#3	Lung adenocarcinoma	Yes	4.6	Yes
#4	Lung adenocarcinoma	No	8.2	Yes
#5	Histiocytic sarcoma	Yes	11.3	Yes
#6	Metastases of malignant epithelial tumor	Yes	18.3	Yes

CT, computed tomography.

## 4. Discussion

All pulmonary tumors showed the ICG fluorescence irrespective of histopathological diagnosis; therefore, it was possible to intraoperatively identify the degree of infiltration of pulmonary tumors. However, most masses were lung adenocarcinoma, with only a few other lesion types in our study. Therefore, further clinical studies are required to clarify the usefulness of ICG fluorescence in differential diagnosis of lung tumors.

The mechanism by which ICG fluoresces in tumors remains unclear in the field of human medicine. It is believed that anion-transporting peptides, intracellular transporters, and export transporters are involved in the mechanism by which hepatic tumors exhibit ICG fluorescence in human medicine (22). In contrast, the enhanced permeability and retention (EPR) effect has been suggested as the mechanism by which tumors other than hepatic tumors exhibit ICG fluorescence (23). The EPR effect is a property by which small molecules such as ICG, which are injected systemically and passively, accumulate in tumors due to the presence of defective endothelial cells and wide fenestrations (600–800 nm) in nascent blood vessels (24). In our study, the accumulation of ICG particles in tumors due to the EPR effect was thought to be responsible for fluorescence in all tumors. Fluorescence was also observed in non-tumor, macrophage-associated nodules; however, tumor and inflammatory tissues have been reported to undergo similar vascular changes in the microenvironment (25). A phenomenon similar to the EPR effect may have occurred in this nodule showing fluorescence. Nevertheless, it is necessary to investigate the fluorescence mechanism of ICG in pulmonary tumors in dogs.

Our study demonstrated a low diagnostic accuracy for the complete resection of lung tumors in dogs. Since there were only a few cases in which the fluorescence of the excised margin was evaluated, it is necessary to further increase the sample size in future studies. Furthermore, the percentage of false positives (cases in which fluorescence was observed at the resection margin despite being a complete resection) was high. In humans, a previous study reported that ICG is metabolized in the liver, resulting in shorter visualization times in the lungs (26). Moreover, it has been reported that ICG can be administered intravenously at 1–5 mg/kg for the fluorescence imaging of human pulmonary tumors (27–29). The maximum dose of ICG for intravenous injection in humans is set at 5.0 mg/kg (30–34). In addition, the occurrence rate of allergic reactions with doses below 0.5 mg/kg was reported as 0.003%, which increased significantly when the dose exceeded 5.0 mg/kg (35). In a previous study, 5.0 mg/kg of ICG was administered for the fluorescence imaging of canine pulmonary tumors (36). No adverse event was observed at the ICG dose of 2.0 mg/kg used in our study. However, further investigations are required to establish the optimal dose of ICG for fluorescence imaging in canine pulmonary tumors. Determining the optimal ICG dose may improve its sensitivity and specificity in the evaluation of the complete resection.

Our study revealed that the presence of lymph node metastasis could be determined with a high probability by assessing ICG fluorescence observation of the lymph nodes. When lymph node fluorescence was observed, there was a tendency for the size of the lymph node on CT to increase. A previous study reported that metastasis to tracheobronchial lymph nodes can be evaluated by CT imaging with a sensitivity of 85.7% and specificity of 95.2% in dogs (21); however, when measured under the same conditions in the present study, the sensitivity was low. In lymph node metastasis of lung tumors in dogs, the ICG fluorescence method was sensitive, whereas a previous study reported that CT had high specificity (21). Therefore, better results might be obtained by combining these two methods. However, our study did not conclude that the combination of ICG fluorescence and CT imaging may be useful because of the low sensitivity of CT for lymph node metastasis. There was no significant relationship between the size of tracheobronchial lymph nodes in the CT imaging and the presence of lymph node metastasis, while there was a significant relationship between ICG fluorescence of the lymph nodes and the presence of lymph node metastasis. In human medicine, ICG fluorescence has also been used for sentinel lymph node mapping in pulmonary tumors (37). The identification rate of sentinel lymph nodes for human pulmonary tumor using isosulfanblue is low (22–50%) (37). In contrast, ICG administered intraoperatively around the tumor has been reported to have an identification rate of 80.7% (38). In our study, ICG was administered intravenously before surgery. Intravenously administered ICG is thought to be moved into the tumor cells by the EPR effect and then flow into the lymphatic vessels, which may produce the ICG fluorescence in the regional lymph nodes. Our study suggests that, like intraoperative peri-tumoral injection of ICG, intravenous administration of ICG can be used for sentinel lymph node mapping of canine lung tumors. However, our study did not investigate the difference in fluorescence between intraoperative peri-tumoral and preoperative intravenous administration of ICG. Therefore, further studies are warranted to clarify the optimal route of ICG administration for the intraoperative identification of lung tumor and sentinel lymph node mapping in dogs.

The limitations of our study include the small number of cases in which the intraoperative and postoperative conditions of the dogs allowed evaluation of the fluorescence of the resected margins and lymph nodes. The suitable dose and duration of ICG administration are still controversial.

In conclusion, ICG fluorescence imaging might be a useful intraoperative diagnostic method to identify tumor location and lymph node metastasis, but not for evaluation of the complete resection in dogs with pulmonary malignant tumors.

## Data availability statement

The original contributions presented in the study are included in the article/supplementary material, further inquiries can be directed to the corresponding author.

## Ethics statement

The animal study was reviewed and approved by Ethical Committee of Nihon University Animal Medical Center. Written informed consent was obtained from the owners for the participation of their animals in this study.

## Author contributions

KA and KI contributed to designing and planning the study, participated in the surgeries and peri-operative management, wrote, and reviewed the manuscript. NS contributed to designing and planning the study, participated in the surgeries and peri-operative management, and wrote the manuscript. KT, TH, and OY contributed to supporting the study and participated in the surgeries and peri-operative management. YK and KO contributed to evaluating histopathological examination. All authors contributed to the article and approved the submitted version.
